# A comprehensive rhythmicity analysis of host proteins and immune factors involved in malaria pathogenesis to decipher the importance of host circadian clock in malaria

**DOI:** 10.3389/fimmu.2023.1210299

**Published:** 2023-08-10

**Authors:** Sourbh Rankawat, Kavita Kundal, Shreyayukta Chakraborty, Rahul Kumar, Sandipan Ray

**Affiliations:** Department of Biotechnology, Indian Institute of Technology Hyderabad, Kandi, Telangana, India

**Keywords:** circadian rhythm, immune factors, infection-immunity-clock, host immune response, malaria, *Plasmodium*

## Abstract

**Background:**

Circadian rhythms broadly impact human health by regulating our daily physiological and metabolic processes. The circadian clocks substantially regulate our immune responses and susceptibility to infections. Malaria parasites have intrinsic molecular oscillations and coordinate their infection cycle with host rhythms. Considering the cyclical nature of malaria, a clear understanding of the circadian regulations in malaria pathogenesis and host responses is of immense importance.

**Methods:**

We have thoroughly investigated the transcript level rhythmic patterns in blood proteins altered in falciparum and vivax malaria and malaria-related immune factors in mice, baboons, and humans by analyzing datasets from published literature and comprehensive databases. Using the Metascape and DAVID platforms, we analyzed Gene Ontology terms and physiological pathways associated with the rhythmic malaria-associated host immune factors.

**Results:**

We observed that almost 50% of the malaria-associated host immune factors are rhythmic in mice and humans. Overlapping rhythmic genes identified in mice, baboons, and humans, exhibited enrichment (Q < 0.05, fold-enrichment > 5) of multiple physiological pathways essential for host immune and defense response, including cytokine production, leukocyte activation, cellular defense, and response, regulation of kinase activity, B-cell receptor signaling pathway, and cellular response to cytokine stimulus.

**Conclusions:**

Our analysis indicates a robust circadian regulation on multiple interconnected host response pathways and immunological networks in malaria, evident from numerous rhythmic genes involved in those pathways. Host immune rhythms play a vital role in the temporal regulation of host-parasite interactions and defense machinery in malaria.

## 1 Introduction

Circadian rhythm is a biological mechanism that governs the cyclic variations in a wide diversity of physiological, metabolic, immunological, and behavioral processes over approximately 24 hours (1, 2). The circadian clock tightly regulates our immune system, including the functioning of natural killer cells, neutrophils, monocytes, eosinophils, and macrophages, most likely to provide a steady defense in accordance with diurnal peaks of pathogenic infections (3, 4). Cellular immunity against pathogenic microbes temporally controlled by the core clock circuit leads to fluctuations in our susceptibility to infections at different times of the day (5). In this vein, multiple studies have demonstrated that the time of day of infection has a vital influence on the progression and severity of infectious diseases (6–8).

The cyclical nature of malaria is well documented, as the infection cycles for *Plasmodium* species that infect humans last a multiple of approximately 24 hours (9). Importantly, recent studies have demonstrated that malaria parasites have intrinsic molecular oscillations and effectively synchronize their rhythms with the host (10, 11). Malaria parasites control the intra-erythrocytic developmental cycle (IDC) progression through coordination with host timekeeping machinery (12). Synchronous maturation of *Plasmodium in vivo* is coordinated and regulated by the rhythmic changes in melatonin concentration in our body (13). If malaria parasites’ cell cycle timing is perturbed relative to the host’s circadian rhythm, it causes reduced replication and transmission of the pathogen (14, 15).

**Figure 1:**
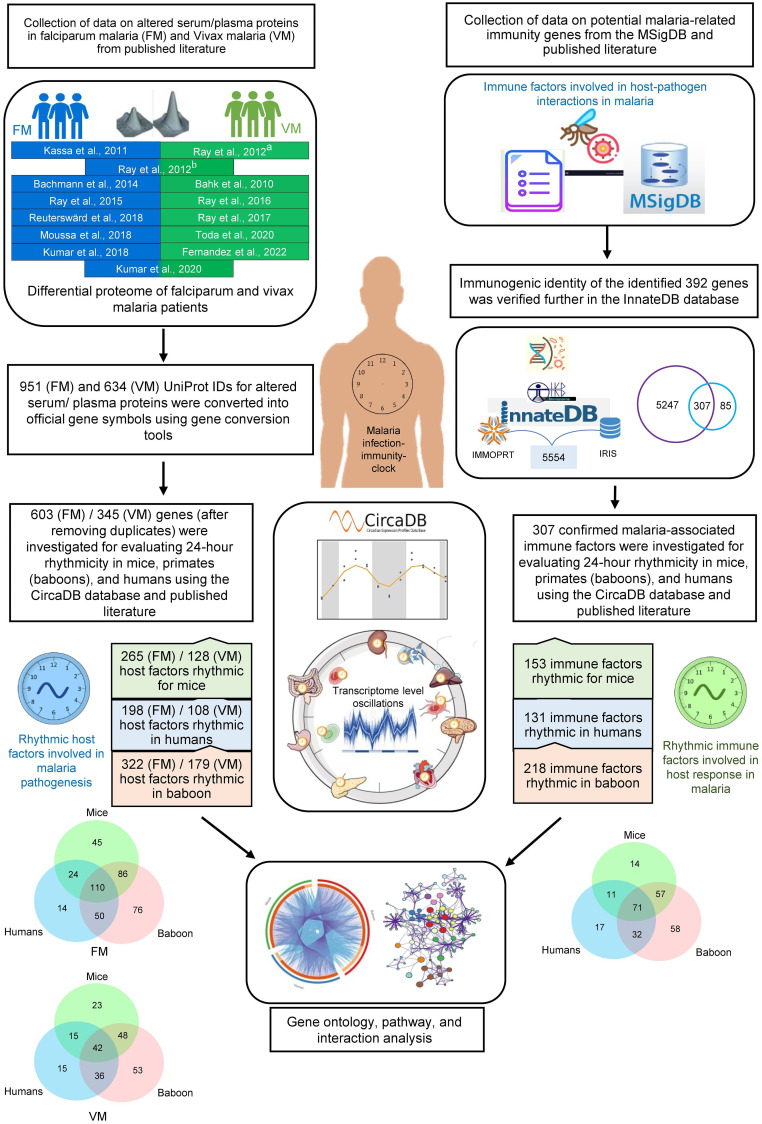
Schematic showing the analysis pipeline for investigating rhythmicity of host proteins and immune factors involved in malaria pathogenesis. At the onset, we collected data on altered serum, plasma, and tissue proteins in falciparum and vivax malaria and malaria-related immunity genes from published literature and databases. Consequently, we investigated 24-hour rhythmicity in mice, primates (baboons), and humans using the CircaDB database and published literature. Further, we performed Gene Ontology, pathway, and interaction analysis on the rhythmic host proteins and immune factors involved in malaria pathogenesis to interpret the importance of the host circadian clock in malaria.

Among the five species of *Plasmodium* that can infect humans and cause malaria, *Plasmodium falciparum and Plasmodium vivax* are the primary pathogens of this protozoan infection. Severe *P. falciparum* and *P. vivax* infections alter many vital physiological pathways, including cytokine signaling, complement systems, inflammation, platelet hemostasis, and coagulation cascades in humans (16–19). Various pathogens, including the malaria parasites, disrupt host circadian rhythms as a virulence trait to favor their replication and dissemination (20). Robust circadian timekeeping machinery in the host may reduce vulnerability towards any acute infections or severity of their clinical manifestations. This study systematically investigated the rhythmic patterns in serum, plasma, and tissue proteins altered in malaria and malaria-related immune factors by analyzing data from published literature and comprehensive databases (**Figure 1**). We also mapped the diurnal rhythmicity in vital physiological pathways and host immunity cascades involved in malaria. Our findings indicate that several components within the host physiological pathways and immunological networks activated in malaria exhibit robust 24-hour rhythms. A detailed understanding of host rhythms may open new opportunities for developing clock-based efficient prophylactic and therapeutic strategies for malaria.

## 2 Methods

### 2.1 Documentation of host proteins altered in falciparum and vivax malaria

Information on differentially abundant serum or plasma proteins in two primary clinical forms of malaria, *P. falciparum* and *P. vivax* infections was retrieved from relevant published literature (16, 17, 19, 21–30) (**Supplementary Tables 1**, **2**). We also included a study describing the altered proteome of the frontal lobe in cerebral falciparum malaria patients (18). Human proteins altered in non-severe and severe falciparum and vivax malaria patients were recorded for subsequent analyses (**Figure 1**). UniProt IDs of the altered proteins from the published literature were converted into official gene symbols using the Database for Annotation, Visualization, and Integrated Discovery (DAVID) gene conversion tool (https://david.ncifcrf.gov/home.jsp) (31).

### 2.2 Cataloging of immunity-related genes in malaria

Genes involved in immune responses in malaria were searched using the keywords “Malaria immunity genes,” “Host immune response in malaria,” and “Host defense mechanism in malaria” in PubMed and Google Scholar. Moreover, potential malaria-related immunity genes were searched in the Molecular Signatures Database (MSigDB) (https://www.gsea-msigdb.org/gsea/msigdb/index.jsp) using the keyword “*Plasmodium*.” MSigDB is an extensively used, wide-ranging database of genes for the execution of gene set enrichment analysis (32). We removed the duplicates after combining the two data sets (entities from the published literature and MSigDB). A list of 392 malaria-related immunity genes was generated for further validation (**Supplementary Table 3**). The immunogenic identity of the identified 392 genes was verified further in the InnateDB database (https://www.innatedb.com) (**Figure 1**). InnateDB is a comprehensive centralized resource for immune function-related genes (33). Venny 2.1 was used to analyze overlaps among different datasets (https://bioinfogp.cnb.csic.es/tools/venny/).

### 2.3 Rhythmicity analysis of the malaria-associated host factors

Circadian expression profile database (CircaDB) (http://circadb.hogeneschlab.org/) was used as a comprehensive resource to investigate the rhythmicity of the malaria-associated immunity genes and host factors in mice and humans. CircaDB provides information on the transcript level oscillations in various tissues of mice and humans (34, 35). Genes with robust oscillations in humans and mice were defined using period length and FDR thresholds (Period 24 ± 3 hours; JTK Q < 0.1). Rhythmicity of the malaria-associated immunity genes and host factors in a primate (baboon, *Papio anubis*)” was investigated in the diurnal transcriptomics datasets reported by Mure et al. (36). The study analyzed the diurnal transcriptome profiles in 64 tissues collected every 2 hours over a 24-hour cycle, where a *p*-value < 0.05 (MetaCycle algorithm) statistical threshold was used to define rhythmicity. Subsequently, the rhythmic malaria-associated immunity genes and host factors were compared in these three mammals (humans, mice, and baboons), and their overlaps were visualized in a Circos plot (37). We also used Venny 2.1 to analyze overlaps among three datasets (humans, mice, and baboons) and plot the Venn diagrams.

### 2.4 Gene Ontology and pathway enrichment analysis

The pathway and Gene Ontology (GO)-Biological Process (BP) enrichment analysis of the rhythmic genes was carried out using the Metascape platform (https://metascape.org/gp/index.html), which combines multiple databases (GO Biological Processes, Kyoto encyclopedia of genes and genomes (KEGG) pathway, Reactome gene sets, Comprehensive resource of mammalian protein complexes (CORUM), WikiPathways, and Protein analysis through evolutionary relationships (PANTHER) pathway (38). The top network clusters were generated by choosing terms based on *p*-value, enrichment factor, and minimum count thresholds. Grouped terms with a similarity > 0.3 were connected by edges and visualized using Cytoscape (https://cytoscape.org/) (39). Further, using the DAVID database, we examined the KEGG pathways of the overlapping genes (40). We visualized the physiological pathways associated with the rhythmic genes through a bubble plot representation using the SR Plot platform (https://www.bioinformatics.com.cn/en).

Enrichment of transcription regulator using the Transcriptional regulatory relationships unraveled by sentence-based text mining (TRRUST) database (https://www.grnpedia.org/trrust/), and based on kappa-statistical similarities, significant terms were hierarchically clustered into a tree. TRRUST is a comprehensive reference database of human transcriptional regulatory networks (41). A threshold of 0.3 kappa score was applied to divide the tree into term clusters, and the lowest *p*-value was selected to visualize the top 20 terms into a heatmap/dendrogram.

### 2.5 Protein-protein interaction network analysis

Protein-protein interaction (PPI) enrichment analysis was performed utilizing the Metascape platform using the following databases (38): Search tool for the retrieval of interacting genes/proteins (STRING), Biological general repository for interaction datasets (BioGrid), OmniPath, and InWeb-IM. Proteins that form physical interactions following the default analysis parameters with at least one other member of the list were used in the analysis (38). The Molecular complex detection (MCODE) algorithm was applied using best-scoring by *p*-value threshold to identify densely connected network components (42). The final cluster was visualized by Cytoscape using Y-files radial layout (39).

## 3 Results

### 3.1 Rhythmic patterns in serum, plasma, and tissue proteins altered in falciparum malaria

**Figure 2:**
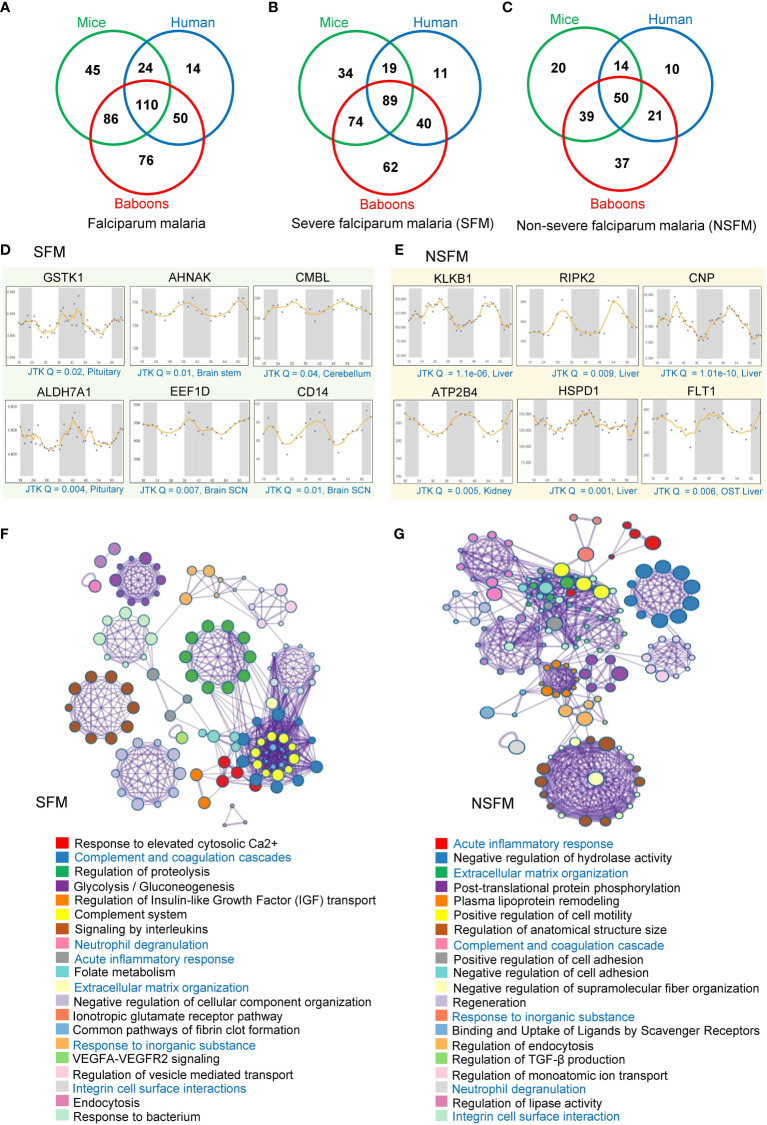
Rhythmic host proteins and physiological pathways altered in falciparum malaria. **(A)** Venn diagram showing serum and plasma proteins altered in falciparum malaria patients (including SFM and NSFM) that exhibit 24-hr rhythmicity across mice, humans, and primates under healthy conditions. **(B, C)** Venn diagrams showing rhythmic host proteins altered in SFM **(B)** and NSFM **(C)**, representing their distribution across mice, humans, and primates. **(D, E)** Transcript level profiles (in different organelles of mice for two day-night cycles) of a few representative highly rhythmic host proteins (JTK Q < 0.05, period 24 ± 3 hours) that are altered in SFM **(D)** and NSFM **(E)**. The X-axis represents time (h), while the Y-axis indicates normalized intensity for transcripts. We used the Circadian Expression Profiles Database (CircaDB) (http://circadb.hogeneschlab.org/) for rhythmicity analysis. **(F, G)** The top 20 statistically enriched GO/KEGG terms and canonical pathways (*p*-value < 0.01, EF > 1.5, Count > 5) associated with the rhythmic host proteins identified in SFM **(F)** and NSFM **(G)** obtained through Metascape analysis.

We analyzed possible rhythmic patterns of 603 differentially abundant proteins in falciparum malaria (FM) at the transcription level using the CircaDB platform (**Supplementary Table 1**). Among these 603 malaria-associated host factors, we identified 198 (32.8%) and 265 (43.9%) rhythmic candidates (Period 24 ± 3 h, JTK Q < 0.1) in humans and mice, respectively. Using a diurnal transcriptome dataset (36), we also investigated the expression patterns of these malaria-associated host factors in the baboon. Non-human primates models are vital to malaria research for understanding its pathophysiology. We identified 322 (53.3%) rhythmic malaria-associated host factors in the primate model (**Figure 2A**). Furthermore, we conducted a comparative analysis of rhythmic candidates identified in severe falciparum malaria (SFM) and non-severe falciparum malaria (NSFM); we identified 329 rhythmic candidates in SFM and 191 rhythmic candidates in NSFM across mice, humans, and primates (**Supplementary Table 1**). We identified 216 (42.4%) rhythmic candidates in mice, 159 (31.3%) in humans, and 265 (52.2%) in primates in SFM (**Figure 2B**, **Supplementary Table 1**). Similarly, in NSFM, we found 123 (39.8%) rhythmic candidates in mice, 95 (30.7%) in humans, and 147 (47.5%) in primates (**Figure 2C**, **Supplementary Table 1**). Further, we compared the rhythmic malaria-associated host factors in mice, humans, and primates in SFM and NSFM to identify overlapping rhythmic candidates between the three organisms. The result indicates 89 overlapping rhythmic candidates in SFM and 50 overlapping rhythmic candidates in NSFM (**Figures 2B, C**). **Figures 2D, E** displays the transcript-level profiles of the selected top rhythmic host factors altered in SFM and NSFM. Robust transcript level oscillations (JTK Q < 0.05) of these genes are observed in the liver, brain, and kidney, which are affected in complicated FM.

### 3.2 Rhythmicity in multiple vital physiological pathways associated with host factors altered in falciparum malaria

We investigated the physiological pathways associated with rhythmic malaria-associated host factors in mice, baboons, and humans, encompassing SFM and NSFM. Employing Metascape analysis, we examined the rhythmic candidates specific to SFM and NSFM to understand the dysregulated pathways with malaria severity. Analysis of rhythmic host proteins identified in SFM revealed enrichment of multiple physiological pathways vital for host-pathogen interaction and immune response, including complement and coagulation cascades (Q = 6.04E-12, fold enrichment (FE) = 27), signaling by interleukins (Q = 7.21E-09, FE = 7), regulation of proteolysis (Q = 3.37E-09, FE = 6), response to elevated platelet cytosolic Ca2+ (Q = 6.04E-12, FE = 20), and complement System (Q = 6.25E-09, FE = 20) (**Figure 2F**, **Supplementary Table 4**).

A similar analysis of rhythmic host proteins identified in NSFM revealed enrichment of acute inflammatory response (Q = 0.0001, FE = 19), post-translational protein phosphorylation (QE = 0.0002, FE = 10), plasma lipoprotein remodeling (Q = 0.001, FE = 24), extracellular matrix organization (Q = 0.0003, FE = 10.6) and negative regulation of hydrolase activity (Q = 0.0001, FE = 7) and other pathways relevant to malaria pathogenesis (**Figure 2G**, **Supplementary Table 4**). Notably, we observed complement and coagulation cascades, neutrophil degranulation, acute inflammatory response, response to inorganic substance, extracellular matrix organization, and integrin cell surface interactions as overlapping pathways associated with the rhythmic host proteins identified in both SFM and NSFM (**Figures 2F, G**).

### 3.3 Analysis of rhythmic patterns in serum and plasma proteins altered in vivax malaria

**Figure 3:**
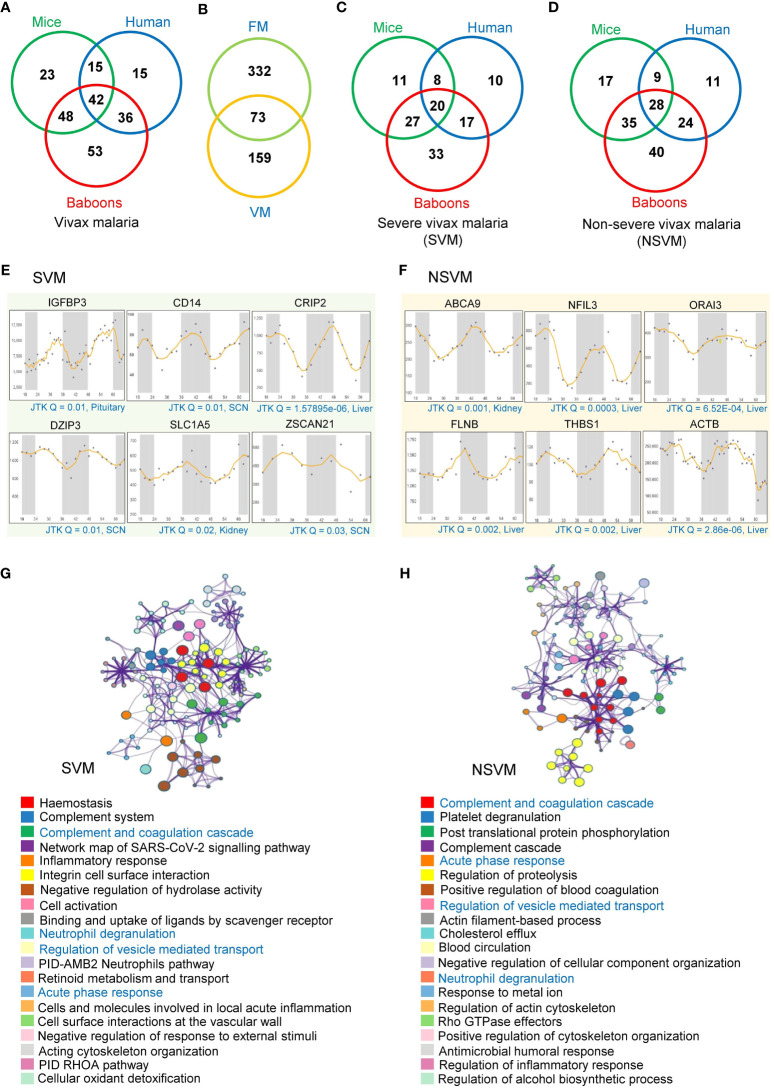
Rhythmic host proteins and their associated pathways altered in vivax malaria. **(A)** Venn diagram showing serum and plasma proteins altered in vivax malaria patients (including SVM and NSVM) that exhibit 24-hr rhythmicity across mice, humans, and primates under healthy conditions. **(B)** Overlap between the rhythmic host proteins identified in FM and VM. **(C, D)** Venn diagrams showing rhythmic host proteins altered in SVM **(C)** and NSVM **(D)**, depicting their distribution across three mammalian species (mice, humans, and primates). **(E, F)** Transcript level profiles (in different organelles of mice for two day-night cycles) of a few representative highly rhythmic host proteins (JTK Q < 0.05, period 24 ± 3 hours) that are altered in SVM **(E)** and NSVM **(F)**. The X-axis represents time (h), while the Y-axis indicates normalized intensity for transcripts. CircaDB (http://circadb.hogeneschlab.org/) was used for rhythmicity analysis. **(G, H)** The top 20 statistically enriched GO/KEGG terms and canonical pathways (*p*-value < 0.01, EF > 1.5, Count > 5) associated with the rhythmic host proteins identified in SVM **(G)** and NSVM **(H)**.

We also investigated the rhythmic patterns of 345 serum or plasma proteins that exhibited differential abundance in vivax malaria (VM) (**Supplementary Table 2**). We identified 128 (37%) and 108 (31.3%) rhythmic candidates (Period 24 ± 3 h, JTK Q < 0.1) among these malaria-associated host factors in mice and humans, respectively. Moreover, our analysis revealed 179 (51.8%) rhythmic malaria-associated host factors in primates (**Figure 3A**). Subsequently, we compared the rhythmic human proteins identified in FM and VM, which indicated 73 overlapping rhythmic candidates (**Figure 3B**).

Alike FM, we precisely investigated the rhythmic host proteins in different severity levels of VM. We identified 126 rhythmic candidates in severe vivax malaria (SVM), while non-severe vivax malaria (NSVM) was associated with 164 rhythmic candidates (**Figures 3C, D**). Cross-comparing the rhythmicity data across three species, i.e., mice, humans, and baboons, we identified 20 and 28 overlapping rhythmic candidates in SVM and NSVM, respectively. We have shown the rhythmic expression profiles of a few representative transcripts in SVM and NSVM, robustly oscillating in mice, humans, and baboons (**Figures 3E, F**).

### 3.4 Physiological pathways associated with the rhythmic host factors altered in vivax malaria

We intended to map the various physiological pathways associated with the rhythmic host factors identified in SVM and NSVM (**Supplementary Table 4**). In SVM, the key enriched pathways includes hemostasis (Q = 2.80E-11, FE = 13.9), the complement and coagulation cascades (Q = 5.78E-08, FE = 44.3), inflammatory response (Q = 7.04E-07, FE = 10.8), PID-AMB2 neutrophil pathway (Q = 5.32E-04, FE = 55.5), and integrin cell surface interactions (Q = 7.93E-06, FE = 35) (**Figure 3G**). In NSVM, we observed that the rhythmic host factors are primarily involved in platelet degranulation (Q = 3.3E-08, FE = 23), blood coagulation (Q= 3.44E-04, FE = 39), proteolysis (Q = 2.05E-04, FE = 4.8), post-translational protein phosphorylation (Q = 1.28E-06, FE = 19.94), and humoral responses (Q = 4.85E-02, FE = 7.12) (**Figure 3H**). Four pathways, namely complement and coagulation cascades, neutrophil degranulation, regulation of vesicle-mediated transport, and the acute-phase response, were common between SVM and NSVM.

Inclusive analysis of all the rhythmic host proteins altered in FM and VM indicated enrichment of complement and coagulation cascades, neutrophil degranulation, and vascular endothelial growth factor A (VEGFA) -VEGF receptor 2 (VEGFR2) signaling as the most significant entities (**Table 1**). The circadian clock tightly regulates these pathways under healthy conditions, and blood levels of several components of these pathways are altered in *P. falciparum and P. vivax* infections.

**Table 1:** Rhythmic host proteins associated with physiological pathways altered in falciparum and vivax malaria*.

Sl No.	Gene ID**	Gene Name	Rhythmicity in mammalian tissues or organs***
Complement and Coagulation Cascades			
1	*A2M^a^*	Alpha-2-macroglobulin	Cerebellum (JTK-Q = 4.94E-02)
2	*FGA^a^*	Fibrinogen alpha chain	Liver (JTK-Q = 5.07E-04)
3	*ITIH3^a^*	Inter-alpha-trypsin inhibitor heavy chain 3	Liver (JTK-Q = 6.87E-03)
4	*SERPINC1^a^*	Serpin family C member 1	Liver (JTK-Q = 2.70E-03)
5	*ATP2B4^b^*	ATPase plasma membrane Ca2+ transporting 4	Cerebellum (JTK-Q = 4.30E-02)
6	*KLKB1^b^*	Kallikrein B1	Liver (JTK-Q =1.10E-06)
7	*SERPIND1^b^*	Serpin family D member 1	Liver (JTK-Q = 6.31E-03)
8	*F10 ^b^*	Coagulation factor X	SCN (JTK-Q = 2.18E-02)
9	*F11^b^*	Coagulation factor XI	Liver (JTK-Q = 3.92E-02)
10	*F5^b^*	Coagulation factor V	Liver (JTK-Q = 4.57E-02)
11	*F9^b^*	Coagulation factor IX	Liver (JTK-Q = 2.25E-02)
12	*FGB^b^*	Fibrinogen beta chain	Liver (JTK-Q = 4.01E-02)
13	*LGALS3BP^b^*	Galectin-3-binding protein	Liver (JTK-Q = 2.11E-02)
14	*OLA1^b^*	Obg-like ATPase 1	Liver (JTK-Q = 2.31E-03)
15	*PPIA^b^*	Peptidyl-prolyl cis-trans isomerase A	Cerebellum (JTK-Q = 7.53E-03)
16	*SERPINE1^b^*	Serpin family E member 1	Liver (JTK-Q = 3.15E-02)
17	*SERPINE2^b^*	Serpin family E member 2	Liver (JTK-Q = 1.10E-06)
18	*TUBA1C^b^*	Tubulin Alpha 1c	Liver (JTK-Q = 2.11E-02)
19	*TUBA4A^b^*	Tubulin Alpha 4a	Liver (JTK-Q = 2.50E-11)
20	*TUBB2A^b^*	Tubulin beta 2A class IIa	Liver (JTK-Q = 1.02E-03)
21	*ACTB^c^*	Actin, cytoplasmic 1	Liver (JTK-Q = 8.07E-08)
22	*WDR1^c^*	Wd repeat domain 1	SCN (JTK-Q = 1.78E-02)
23	*GLG1^c^*	Golgi glycoprotein 1	SCN (JTK-Q = 1.78E-02)
24	*VCL^c^*	Vinculin	SCN (JTK-Q = 6.51E-03)
Neutrophil Degranulation			
1	*CST3^a^*	Cystatin C	Cerebellum (JTK-Q = 4.30E-02)
2	*HSPA8^a^*	Heat shock protein family A (hsp70) member 8	Liver (JTK-Q = 5.85E-03)
3	*CTSD^b^*	Cathepsin D	SCN (JTK-Q = 1.35E-02)
4	*EEF2^b^*	Eukaryotic translation elongation factor 2	SCN (JTK-Q = 3.93E-02)
5	*FTH1^b^*	Ferritin heavy chain 1	SCN (JTK-Q = 2.92E-03)
6	*ITGAV^b^*	Integrin subunit alpha V	SCN (JTK-Q = 6.51E-03)
7	*PGAM1^b^*	Phosphoglycerate mutase 1	Liver (JTK-Q = 4.74E-03)
8	*PPIA^b^*	Peptidyl-prolyl cis-trans isomerase A	Cerebellum (JTK-Q = 7.53E-03)
9	*PRDX6^b^*	Peroxiredoxin-6	Liver (JTK-Q = 6.87E-03)
10	*PSMB1^b^*	Proteasome subunit beta type-10	SCN (JTK-Q = 1.93E-02)
11	*PSMB7^b^*	Proteasome 20s subunit beta 7	Liver (JTK-Q =1.72E-02)
12	*PIGR^c^*	Polymeric immunoglobulin receptor	Liver (JTK-Q = 1.62E-02)
13	*VCL^c^*	Vinculin	SCN (JTK-Q = 6.51E-03)
14	*VNN1^c^*	Vanin 1	Liver (JTK-Q = 1.42E-04)
15	*FLG2^c^*	Filaggrin 2	SCN (JTK-Q = 3.16E-02)
VEGFA-VEGFR2 Signaling			
1	*CFL1^a^*	Cofilin-1	Liver (JTK-Q = 1.72E-02)
2	*FGA^a^*	Fibrinogen alpha chain	Liver (JTK-Q = 5.07E-04)
3	*FGB^a^*	Fibrinogen beta chain	Liver (JTK-Q = 4.01E-02)
4	*GAPDH^a^*	Glyceraldehyde-3-phosphate dehydrogenase	Liver (JTK-Q = 7.41E-03)
5	*PRDX2^a^*	Peroxiredoxin 2	Liver (JTK-Q = 7.41E-03)
6	*CNP^b^*	2’, 3’-cyclic nucleotide 3’ phosphodiesterase	SCN (JTK-Q = 1.65E-03)
7	*FLT1^b^*	Fms-related receptor tyrosine kinase 1	Liver (JTK-Q = 6.92E-03)
8	*HSPB1^b^*	Heat shock protein family B (small) member 1	Cerebellum (JTK-Q = 1.46E-02)
9	*ITGAV^b^*	Integrin subunit alpha V	SCN (JTK-Q = 6.51E-03)
10	*NCL^b^*	Nucleolin	Liver (JTK-Q = 6.16E-06)
11	*PGK1^b^*	Phosphoglycerate kinase 1	Liver (JTK-Q = 1.24E-07)
12	*SOD2^b^*	Superoxide dismutase 2	Liver (JTK-Q = 3.92E-02)
13	*TMOD1^b^*	Tropomodulin 1	Cerebellum (JTK-Q = 5.77E-02)
14	*TUBA1C^b^*	Tubulin Alpha 1c	SCN (JTK-Q = 4.22E-02)
15	*CRIP2^c^*	Cysteine-rich protein 2	Liver (JTK-Q = 1.58E-06)
16	*FGG^c^*	Fibrinogen gamma chain	Liver (JTK-Q = 4.56E-06)
17	*FLNB^c^*	Filamin B	Liver (JTK-Q = 2.29E-03)
18	*VCL^c^*	Vinculin	SCN (JTK-Q = 6.51E-03)

*The most rhythmic host factors (Period 24 ± 3 h, JTK-Q < 0.05) associated with physiological pathways dysregulated in FM and VM (see **Supplementary Tables 1**, **2**, **4** for details).

**Host factors dysregulated in ^(a)^ FM and VM, ^(b)^ FM, and ^(c)^ VM.

***Rhythmicity data are shown for only those mammalian tissues or organs that are associated with malaria pathogenesis.

### 3.5 Rhythmic expression of malaria-related immune factors in mammals

**Figure 4:**
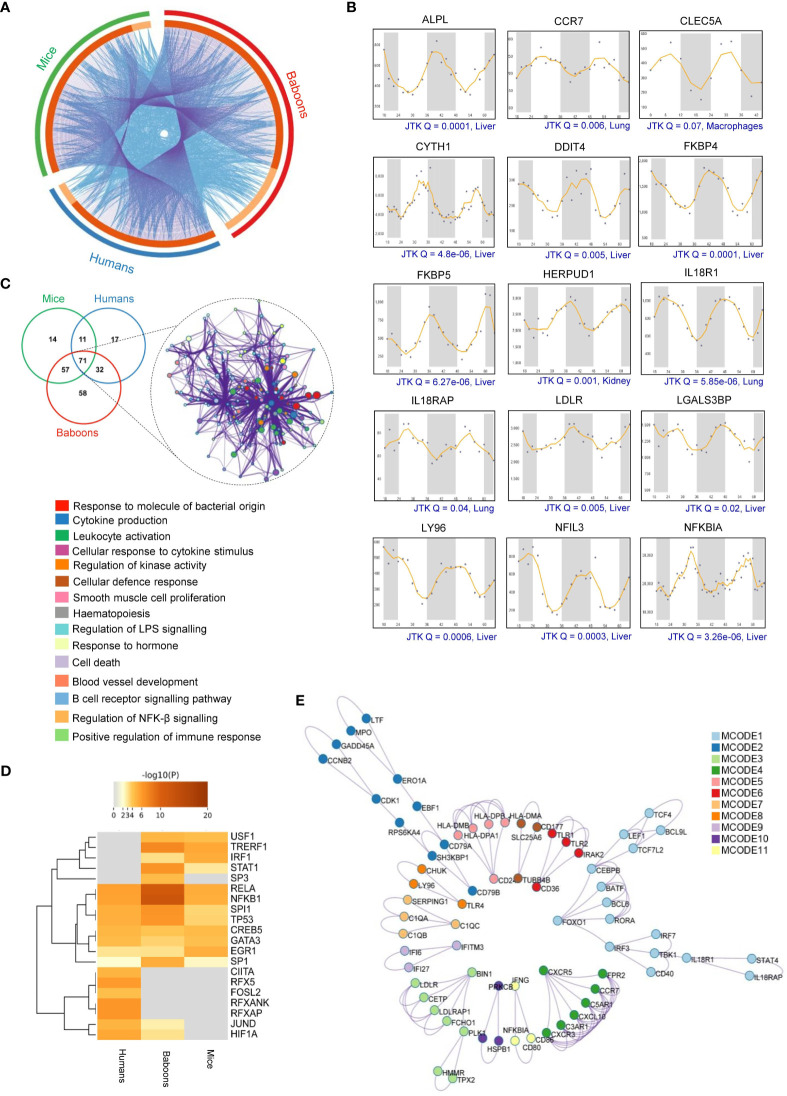
Rhythmicity of malaria-associated immunity genes in mammalian systems. **(A)** The Circos plot shows the overlap of malaria-associated rhythmic immunity genes in mice, primates, and humans. The outer arc represents rhythmic genes specific to mice (green), primates (red), and humans (blue). Dark orange indicates overlapping malaria-associated rhythmic genes in mice, primates, and humans on the inner arc. Light orange indicates malaria-associated rhythmic genes unique to each organism. Blue linker lines represent the same enriched ontology terms, while the purple links represent input gene list overlap. **(B)** Transcript level rhythmic profiles of selected malaria-associated immunity genes in mammalian systems. CircaDB (http://circadb.hogeneschlab.org/) was used for rhythmicity analysis. 24-hour transcript level rhythmic profiles of the immunity genes are shown in different organelles of mice (JTK Q < 0.1, period 24 ± 3 hours). **(C)** Statistically enriched (Top 15, *p*-value < 0.01, Enrichment factor > 1.5, Count > 4) ontology terms (GO/KEGG terms and canonical pathways) associated with the overlapping malaria-associated rhythmic immunity genes identified in mice, primates (baboons), and humans. **(D)** Enrichment analysis of all statistically significant transcription factors (TFs)-target interaction networks associated with malaria-associated rhythmic immunity genes. The heatmap shows the strength of evidence for each TF-target relationship. The intensity of the color represents the strength of the evidence, with brighter colors indicating more robust evidence. **(E)** The protein-protein interaction (PPI) network of the malaria-associated rhythmic immunity genes created by Metascape shows 11 interacting modules (see **Supplementary Table 7** for further details).

Besides the differentially abundant serum or plasma proteins, we investigated the possible rhythmicity in the expression of 307 malaria-associated host immune factors using the CircaDB (**Supplementary Table 3**). We identified 153 (49.84%) rhythmic immune factors (Period 24 ± 3 h, JTK Q < 0.1) in mice and 131 (42.67%) in humans. Further, analyzing the diurnal transcriptome dataset (36), we identified 218 (71.01%) rhythmic malaria-associated host immune factors in baboons. We observed a significant overlap of rhythmic host immunity genes with known biological functions across all three species represented as a Circos plot (**Figure 4A**). Precisely, 71 overlapping rhythmic candidates were identified by comparing the malaria-associated rhythmic host immunity genes of mice, primates, and humans, accounting for 23% of total rhythmic candidates identified in these three species (**Figure 1**). We show here rhythmic expression patterns for several malaria-associated immune factors (top 15 of the 71 overlapping genes) that show almost 24-hour oscillation at the transcript level (JTK Q < 0.1) (**Figure 4B**).

### 3.6 Diurnal rhythmicity in host immunity pathways in malaria

Based on the identified rhythmic malaria-associated host immune factors, we mapped the circadian regulation of diverse immunity pathways that play a crucial role in host response and defense mechanisms in malaria (**Supplementary Table 5**). Pathway analysis involving the 71 overlapping rhythmic immune factors using Metascape revealed the enrichment of multiple physiological pathways essential for host immune and defense response, including cytokine production (Q = 6.00E-04, FE = 12), leukocyte activation (Q = 0.01, FE = 8.9), cellular defense and response (Q = 0.01, FE = 42), regulation of kinase activity (Q = 0.01, FE = 7), B-cell receptor signaling pathway (Q = 0.05, FE = 12), and cellular response to cytokine stimulus (Q = 0.01, FE = 7.7) (**Figure 4C**).

The temporal expression patterns of transcription factor (TF) targets are associated with recurrent patterns observed in many biological processes over a day, including the immune responses. Our analysis of TF-target interactions revealed the enrichment of eight transcription factor-target interactions (RELA, Q = 0.0001, FE= 7.9), NFKB1(Q = 0.00005, FE, 8.2), SPI1(Q = 0.009, FE =15), TP53 (Q = 0.01, FE = 7.9), CREB5 (Q = 0.03, FE = 53), GATA3 (Q = 0.04, FE = 21), EGR1 (Q = 0.03, FE = 12), and SP1(Q = 0.02, FE = 4) that are shared by mice, primates, and humans (**Figure 4D**). These TFs may regulate the temporal expression of host genes related to malaria immunity, suggesting the involvement of a complex molecular network controlled by the timekeeping machinery (**Supplementary Table 6**).

**Figure 5:**
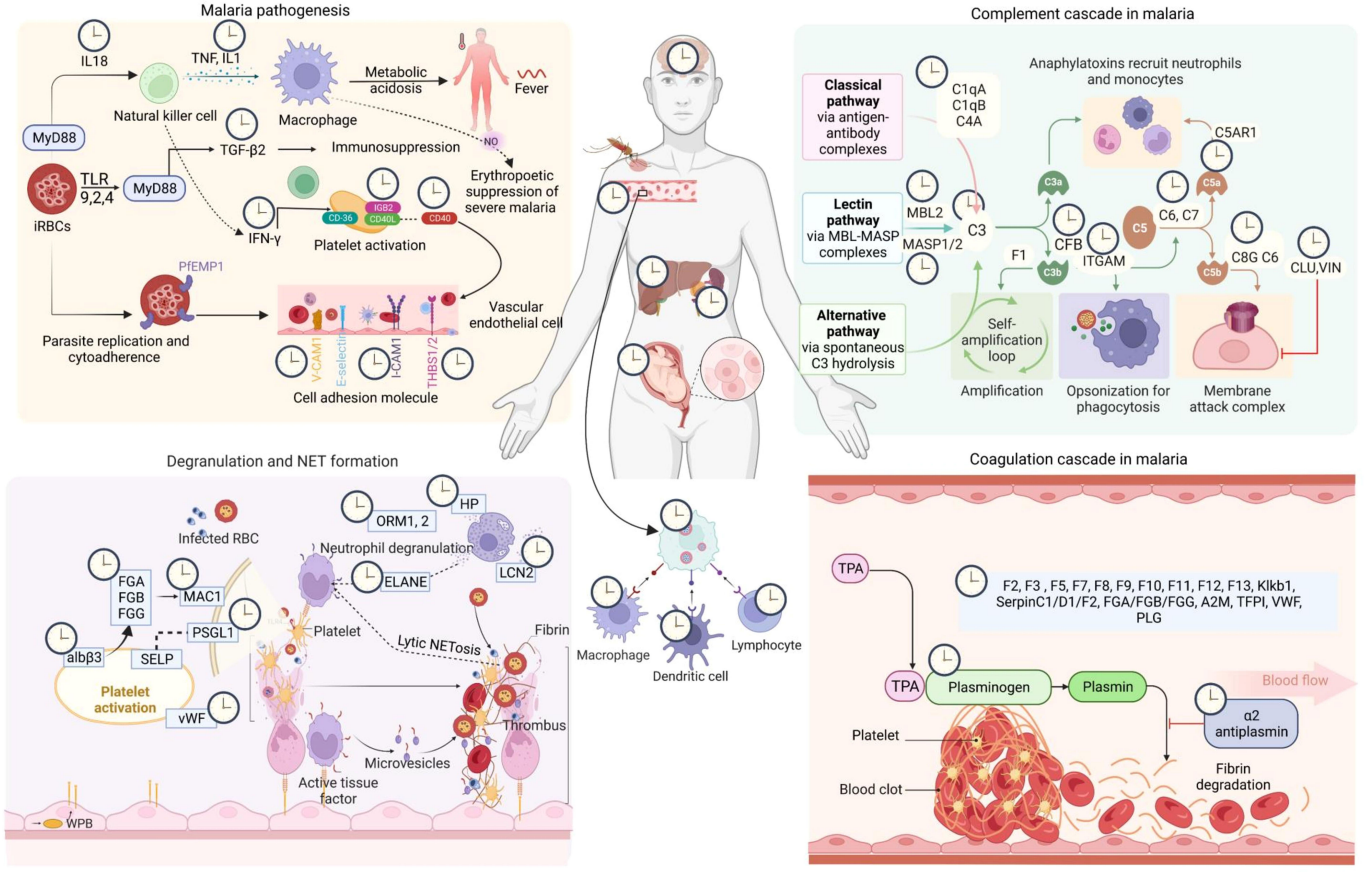
Circadian regulation of host response in malaria. The malaria infection-immunity-clock represents the 24-hour oscillations in host proteins and immune factors that play an essential role in host-pathogen interaction. Here we show circadian regulations of the components of four major host response pathways in malaria (FDR < 0.05, fold enrichment > 2.5, Count > 5). The clock symbols indicate the transcript level rhythmicity (JTK Q < 0.1, period 24 ± 3 hours) of that entity involved in that particular physiological pathway [Created with BioRender.com].

PPI analysis using Metascape and MCODE involving the rhythmic host factors related to malaria-associated immunity in mice, primates, and humans identified 11 PPI modules (**Figure 4E**, **Supplementary Table 7**). Further, for the top three PPI modules based on *p*-values, we applied GO enrichment to the overall MCODE network to get biological relevance from the network component. The top three terms were found to be cell activation (GO:0001775, *p*-value = 1.15E-17), leukocyte activation (GO:0045321, *p*-value = 8.53E-17), and positive regulation of immune response (GO:0050778, *p*-value = 2.83E-16) (**Supplementary Table 7**). Taken together, our analysis indicates that the circadian clock tightly controls multiple interconnected host responses and immunological networks in malaria, which is evident from the presence of several rhythmic components within those pathways (**Figure 5**).

## 4 Discussion

An inadequate understanding of the molecular mechanisms driving complex cyclical pathogenesis and multidimensional fatal manifestations in severe malaria impedes the clinical management of this protozoan infection. Investigation of clock-infection biology can substantially impact various aspects of malaria research, including understanding disease pathogenesis, dynamic host-pathogen interactions, and the time-dependent efficacy of therapeutic interventions. Malaria parasites show developmental rhythms during replication in the mammalian hosts and lead to rhythmic fevers due to the synchronous bursting of the parasite-infected host red blood cells (RBCs) (43). Notably, self-sustained non-canonical redox and metabolic oscillations exist in human RBCs (44, 45). Since our immune system shows rhythmic variations, investigating circadian control in the host response pathways in malaria is vital for understanding the complex pathogenesis of this parasitic infection. Here we report transcript-level rhythmic patterns in malaria-related immune factors and host response pathways in mice, baboons, and humans by analyzing datasets from published literature and wide-ranging databases.

Several physiological pathways controlled by the circadian timekeeping machinery, including complement and coagulation cascades, interleukin signaling, acute inflammatory response, neutrophil degranulation, and VEGFA-VEGFR2 signaling, are dysregulated in malaria. Earlier, we have shown that severe manifestations in *P. falciparum and P. vivax* infections are caused by the modulation of host defense machinery and vital physiological pathways, including cytokine signaling, lipid metabolism, complement pathways, and coagulation cascades, which can be assessed by measuring the alterations in host serum or plasma proteome in malaria patients (16, 17, 19, 46). Other contemporary studies on blood proteome in severe malaria patients also indicated similar alterations in acute phase response, inflammation, and metabolic pathways (21, 24). This study shows that several components of these physiological pathways exhibit robust 24-hour rhythms in both nocturnal and diurnal mammals.

It is critical to decipher whether a circadian disruption in the host contributes to dysregulation in these diverse physiological pathways in malaria. For instance, circadian rhythm firmly controls angiogenesis, potentially by regulating VEGF expression and secretion (47). Notably, the core clock transcription factor brain and muscle ARNT-like-1 (*Bmal1*) regulates the rhythmic expression of VEGF through direct binding to its promoter region (47, 48). Elevated levels of VEGF and VEGFR-2 signaling involved in angiogenesis are frequently observed in individuals suffering from malaria and can serve as a potential biomarker of malaria complications (49, 50). Such alteration in VEGF in malaria is particularly relevant in cerebral manifestations, where the binding of parasitized erythrocytes to the cerebral endothelium and angiogenic dysregulation contributes to the pathogenesis of malaria (49, 51). Thus, circadian disruptions in the host and their subsequent effects on the multidimensional physiological network involved in malaria pathogenesis and complications entail a comprehensive investigation. Rhythmicity information for the elements of host response pathways will help understand possible circadian disruptions in their rhythmic patterns under the febrile or convalescent stages or different severity levels of malaria.

Impairments in liver, brain, and kidney functions in severe *P. falciparum* infection often cause morbidity and mortality in complicated malaria (52–54). *P. vivax* infection also leads to severe malaria involving complicated clinical symptoms, including liver dysfunction, cerebral manifestations, and acute kidney injury (55, 56). Our findings indicate that several malaria-induced altered proteins exhibit 24-hour rhythmicity in the liver, brain, and kidney with high statistical significance (JTK Q < 0.05). Numerous studies have highlighted the crucial role of the rhythmic candidates identified in our analysis in malaria progression and severity. For instance, ADAMTS13 deficiency has been observed in severe malaria, particularly in cerebral manifestations, which may contribute to the pathophysiology of complicated malaria by impairing the regulation of von Willebrand factor (VWF) and promoting endothelial dysfunction (57). KLKB1 involves blood coagulation, inflammation, fibrinolysis, and complement activation (58). The cell adhesion molecule VCAM1 attaches to infected RBCs and is involved in their sequestration (59, 60). SERPINF2 (α2AP) shows altered levels in falciparum malaria patients, contributing to the development of disseminated intravascular coagulation and increasing the risk of thrombosis (19). Additionally, FKBP5, another highly rhythmic candidate in mammals, influences the host immune system and cerebral manifestations of malaria in mice (61, 62).

Host immune factors, cells, and pathways such as pro-inflammatory cascade, cytokine levels, IFN-γ, and natural killer T (NKT) cells determines the disease state, susceptibility, and fatality in malaria (63, 64). Circadian variations in the expression levels of the vital host factors involved in malaria pathophysiology and immunity shown in our study indicate the fluctuations in our defense machinery, which hints at a possible time-dependent vulnerability to the severe manifestations of malaria. Investigating whether disruptions in host circadian rhythms contribute to the severe clinical manifestations frequently observed in malaria patients is an open question. Recently, the “One Health” concept, which emphasizes a trans-disciplinary collaborative approach to strengthen the interrelation between people, animals, and their environment, is gaining popularity in malaria research and elimination programs (65). The circadian aspect of malaria infection biology in the hosts, vector, and pathogen should be emphasized in such a multi-sectoral health strategy.

If validated empirically in preclinical animal models of malaria or malaria patients, our findings can provide novel mechanistic insights into the dynamic host-pathogen interactions in malaria and strengthen the concept of chronotherapeutics for this protozoan infection. In this vein, a systematic investigation of circadian disruptions in non-severe and severe falciparum and vivax malaria patients will be beneficial to decipher if the magnitude of circadian alterations governs the severity of malaria, which could be an imperative continuation of the present study. The rhythmic malaria-related host proteins and immune factors identified in our study could be targeted to understand the overall circadian disruptions in malaria caused by different *Plasmodium* species. Moreover, recent studies have demonstrated the existence of potential markers for peripheral circadian rhythm in biological fluids, such as blood or saliva. Kramer and colleagues have developed a blood-based assay through multiplex gene expression profiling to determine the internal circadian time in humans (66). In another study, β-arrestin 1 (ARRB1) is identified as a molecular marker of the peripheral circadian rhythm in saliva (67). Notably, ∼15% of human plasma and saliva metabolites show circadian rhythmicity (68).

Quantitive analysis of the possible alterations in rhythmicity of proteins and metabolites in blood or saliva samples of malaria patients of different severity levels collected in 3-6 hour resolution for at least two environmental day-night cycles can provide an inclusive representation of dysfunctions in the circadian timekeeping system with malaria severity. Rhythmic molecular markers discussed above could be used as an indicator of a healthy circadian clock to evaluate circadian disruptions in malaria patients. In such case-control longitudinal circadian studies, clock parameters in malaria patients need to be compared against age and gender-matched healthy and febrile controls to provide meaningful information regarding the specificity and adversity of circadian aberrations in core clock machinery and clock-regulated pathways in *Plasmodium* infections.

## 5 Conclusion and limitations

The present study indicates a robust circadian control of various host response pathways and immunological networks that can influence the outcome and severity of clinical manifestations in malaria. We observed a significant overlap among the rhythmic host immunity genes across mice, non-human primates, and humans. Host rhythms are essential for the cyclic regulation of host-parasite interactions and defense machinery in malaria. Understanding host rhythms is critical for developing novel therapeutic strategies for malaria based on boosting the body clocks or correcting circadian disruptions in patients suffering from infection with malaria parasites.

There are a couple of limitations of the present study. The rhythmicity of host immunity factors presented here is analyzed only at the transcript levels based on the available datasets in existing literature and databases. Transcript-level observations do not always reflect rhythmicity at the protein levels, and lower correlations between the rhythmic transcriptome and proteome are observed in many genome-scale circadian studies in different model organisms (69–71). Dysfunctions at the protein levels frequently correlate most accurately with diseased conditions, and most of the existing antimalarial drugs target proteins and metabolic pathways. Consequently, there is a need for protein-level rhythmicity analysis of the malaria-related host factors that we have shown to be involved in multiple interconnected host response pathways and immunological networks. Besides, we have investigated the rhythmicity of malaria-related host factors in circadian transcriptome datasets under healthy conditions. It is essential to study whether those host factors follow a similar cyclic pattern under *Plasmodium* infections. Our present *in silico* study provides an inclusive understanding of 24-hour oscillations in host proteins and immune factors involved in malaria pathogenesis and offers valuable directions for defining experimental strategies for further investigating circadian disruptions in malaria patient cohorts.

## Data availability statement

The original contributions presented in the study are included in the article/**Supplementary Material**. Further inquiries can be directed to the corresponding author.

## Author contributions

Conceptualization: SRay and SRan; Resources: SRay; Data generation and analysis: SRan, SRay, KK, SC, and RK; Writing of the manuscript draft: SRay and SRan. All authors contributed to the article and approved the submitted version.

## Supplementary material

Supplementary Table 1 Rhythmicity analysis (transcript level) of the altered serum, plasma, and tissue proteins in falciparum malaria patients using the Circadian Expression profile database (CircaDB).

Supplementary Table 2 Rhythmicity analysis (transcript level) of the altered serum and plasma proteins in vivax malaria patients using the Circadian Expression profile database (CircaDB).

Supplementary Table 3 List of malaria-associated immunity genes from published literature, MSigDB database, and details for their rhythmicity analysis in mammalian systems using the Circadian Expression Profiles Database (CircaDB).

Supplementary Table 4 Gene Ontology (GO) Biological Process (BP) enrichment analysis of rhythmic (oscillation in mice, primates, and humans; Period 24 ± 3 h, JTK Q < 0.1) in severe and non-severe falciparum and vivax malaria associated rhythmic candidates.

Supplementary Table 5 Pathway and process enrichment analysis of overlapping (in mice, primates, and humans) malaria-associated immunity genes.

Supplementary Table 6 Transcription factor enrichment analysis of rhythmic malaria-associated immunity-related genes.

Supplementary Table 7 Top-ranked Protein-Protein Interactions (PPIs)-enriched clusters for the rhythmic malaria-associated host genes.
